# Insertion-Site Proximity to AAV Inverted Terminal Repeats Increases Plasmid Recombination

**DOI:** 10.3390/ijms27177630

**Published:** 2026-08-26

**Authors:** Maxim Makarenko, Daria Semicheva, Veniamin Fishman

**Affiliations:** 1 Center for Genetics and Life Sciences, Sirius Institute of Science and Technology, Sirius 354340, Russia; makarenko.ms@talantiuspeh.ru (M.M.); semichevadarya@gmail.com (D.S.); 2 Institute of Cytology and Genetics, Novosibirsk 630090, Russia; 3 Department of Natural Sciences, Novosibirsk State University, Novosibirsk 630090, Russia

**Keywords:** adeno-associated virus (AAV), inverted terminal repeats (ITRs), plasmid stability, massively parallel reporter assay (MPRA), recombination

## Abstract

Adeno-associated virus (AAV)-based massively parallel reporter assays (MPRA) have become an important platform for large-scale functional characterization of regulatory DNA elements. However, plasmids carrying AAV inverted terminal repeats (ITRs) are intrinsically unstable during propagation in *Escherichia coli*, potentially compromising library integrity before viral packaging. Although ITR-associated recombination has been recognized, the influence of cloning-site position relative to the ITR on plasmid stability has not been systematically investigated. Here, we examined the relationship between cloning-junction proximity to AAV2 ITRs and plasmid recombination using an AAV-MPRA reporter plasmid. We compared four restriction-ligation cloning strategies utilizing restriction sites at defined distances (4–543 bp) from the nearest ITR while preserving ITR integrity, and one strategy in which the ITR itself was disrupted. We observe that plasmid recombination exhibited a pronounced distance dependence. Constructs with ligation junctions located 4, 41, and 182 bp from an intact ITR showed recombination frequencies of 82.5%, 60%, and 20%, respectively, whereas a 0% recombination frequency was detected when the nearest ITR was positioned 543 bp from the cloning junction. In contrast, cleavage within ITR reduced recombination to 15%, demonstrating that preservation of the intact ITR secondary structure is required for efficient recombination. Whole-plasmid sequencing confirmed recurrent large-scale deletions in which the expression cassette and the downstream R-ITR were removed while the L-ITR and plasmid backbone were retained, consistent with preferential processing of the intact L-ITR region. These findings identify cloning-site proximity to an intact AAV ITR as a major determinant of plasmid stability during bacterial propagation and demonstrate that substantial loss of correctly assembled constructs can occur before AAV production. The results have direct implications for the design of AAV-based MPRA libraries and support positioning cloning sites as far as practical from the nearest ITR, together with routine validation of plasmid integrity prior to viral packaging.

## 1. Introduction

Adeno-associated viruses (AAVs) have emerged as one of the most efficient tools for exogenous DNA delivery in gene therapy. High-efficiency delivery relies on intact ITRs, which the AAV Rep protein uses to initiate genome replication and package the single-stranded genome into the viral capsid. At the same time, AAV ITRs are GC-rich, almost perfectly palindromic sequences that fold into T-shaped hairpins and can form cruciform structures in supercoiled plasmid DNA [1,2]. Such structured repeats are well known to be unstable in bacterial hosts because they are recognized and cut by structure-specific nucleases, leading to deletions and rearrangements within the inverted terminal repeats (ITRs) or the rest of the plasmid [3,4]. While ITR instability is a known hurdle, it becomes especially critical in pooled library approaches, such as massively parallel reporter assays (MPRAs). MPRAs measure the regulatory activity of thousands to hundreds of thousands of candidate DNA elements in a single pooled experiment by cloning each element into a reporter plasmid, linking it to a unique barcode, and quantifying the transcription driven by each element by sequencing its barcode. This approach only works if each barcode stays correctly paired with its original regulatory element throughout all stages of cloning [5,6]. In practice, this pairing can be broken by different recombination-type events during library construction, and even the PCR and packaging steps can produce chimeric barcode–element combinations that distort the readout [7,8]. Since verifying individual clones is impossible in a pooled format, ITR-driven instability poses a severe hidden threat when an ITR-flanked pAAV backbone is used to deliver the reporter library. Currently, two principal library construction strategies are in use, and they differ in how much bacterial amplification occurs in an ITR-containing vector: in one, the barcoded insert pool is assembled and amplified in a standard cloning vector and transferred into the pAAV backbone only at the final step, so that most amplification occurs without ITRs; in the other, the insert pool is cloned directly into the ITR-containing pAAV vector, so that every round of amplification in bacteria occurs in the presence of both ITRs. In both cases, the proximity of the cloning site to the ITR within the final pAAV construct determines how readily ITR-initiated recombination reaches the insert [9,10].

The molecular mechanisms behind this instability are well understood. In supercoiled plasmid DNA, the internal palindromic segment within each ITR can extrude as a cruciform, and when the same region is transiently single-stranded during replication, it can fold back into a hairpin. The bacterial SbcCD nuclease recognizes these unusual DNA shapes and cuts them, producing a double-strand break that is then resected by the RecBCD complex as part of the canonical double-strand break repair pathway in *E. coli* [11,12]. Because this cleavage-and-resection pathway repeatedly targets long inverted repeats, plasmids that retain intact ITRs are strongly counterselected in the population: molecules that survive tend to have the palindrome shortened or disrupted, or to have lost both ITRs. Molecules that escape complete loss often carry smaller deletions, which arise when repeated sequences in and around the ITR misalign during replication and remove part of the hairpin. Long palindromic sequences can also slow or even stall replication forks on both the leading and the lagging strand, which further increases the chance of rearrangements [13]. In line with these mechanisms, standard AAV plasmid preparations often contain mutated or truncated ITRs. For instance, on average about 40% of AAV transfer plasmids carried ITR mutations, preferentially in the 5′ ITR rather than the 3′ ITR, largely due to differences in the GC-rich sequence context flanking each repeat [14]. Recombination-deficient host strains such as Stbl3, SURE, and NEB Stable, together with elevated growth temperatures, can reduce ITR loss but do not eliminate it, and long-read sequencing has shown that the ITR located closer to the plasmid origin of replication tends to degrade faster than the distal ITR [3]. In general, ITR cleavage and repair are ongoing processes during routine plasmid propagation, not rare accidents, and recombination therefore frequently extends beyond the ITR itself into the adjacent backbone. For AAV-MPRA plasmids, this means that any cloning junction placed close to an ITR lies within this damage-and-repair window, so the same events that truncate the ITR can also delete or rearrange the neighboring regulatory cassette and its barcode [15].

Despite this understanding of ITR instability and its mitigations, no study has systematically tested how the distance between the cloning site and the ITR affects recombination frequency in a pAAV-MPRA plasmid. Mechanistically, because ITR-initiated resection and recombination extend into adjacent DNA, an insert placed closer to the ITR should be more likely to be affected than one placed farther away. This question is especially important for AAV-based MPRA because the library is packaged into virus only once and is not re-amplified in bacteria before sequencing; any structural errors introduced during library construction are therefore irreversible and directly carried into the activity readout.

Here we characterize recombination frequency as a function of the distance between the insert’s cloning site and the nearest ITR, using sp8b (4393 bp), a pAAV-based reporter plasmid representative of the standard AAV transfer vector architecture routinely used in our laboratory, as the reference construct. We quantify recombination by colony PCR across five restriction-enzyme cloning strategies that create new ligation junctions at distances of 4–543 bp from the L-ITR, including a construct with direct L-ITR disruption. These strategies were specifically designed for the present study and represent the experimental framework used to test the relationship between cloning-site position and recombinant clone frequency. Although the individual restriction enzyme- and assembly-based cloning procedures are established molecular biology techniques, the specific combinations and junction positions investigated here were designed by the authors. To validate the PCR-based amplicons and dissect the exact molecular architecture of the deletion events, we performed de novo assembly of next-generation sequencing (NGS) data from recombinant clones. Finally, to systematically decouple the effect of the AAV ITR from potential contributions of the cloning method itself, we constructed and analyzed four control libraries on an ITR-free backbone (sp8d) using both Golden Gate and Gibson Assembly, characterized via long-read sequencing.

## 2. Results

### 2.1. Validation of the Restriction–Ligation Construct Panel

The sp8b vector (4393 bp) carries two AAV2 ITRs flanking the reporter cassette: L_ITR_5′ at nt 1–145 and R_ITR_3′ at nt 1652–1796 (Figure S1). Between them lie the chimeric intron (nt 194–326), Kozak sequence, GFP coding sequence (nt 339–1055), and hGH polyA signal (nt 1138–1616). The backbone elements—f1 origin, AmpR, and pUC ori—lie outside the ITR pair, distal to the cassette. This layout is representative of AAV-MPRA reporters in which the candidate regulatory element is cloned between the two ITRs so that it is packaged within the AAV vector genome. We defined the cloning-site-to-ITR distance as the number of base pairs between the nearest restriction cut site (the ligation junction after religation) and the nearest ITR boundary. Using five restriction-enzyme strategies, we created new ligation junctions at distances of 15, 1, 41, 89, and 543 bp from the nearest ITR (Table 1), allowing us to vary the distance variable while holding the backbone sequence constant.

Before evaluating plasmid recombination, we verified that each restriction–ligation strategy generated transformable plasmid molecules. Following restriction digestion, ligation, purification, and electroporation of 1 ng of DNA, all five construct designs produced bacterial colonies, although transformation efficiencies differed among the restriction strategies. The BsaI, EcoRI + KpnI, and KpnI + BtgI constructs yielded approximately 60–100 colonies per transformation, whereas the MluI + EcoRI and BamHI + HindIII constructs generated approximately 200–300 colonies.

As expected, the ligase-only control, in which 100 pg of intact plasmid was transformed without restriction digestion, produced more than 1000 colonies per plate, reflecting the high transformation efficiency of the unmodified plasmid. In contrast, negative control reactions containing a single restriction enzyme in the absence of DNA ligase yielded either no colonies or only occasional single colonies, confirming efficient restriction digestion and demonstrating that colony formation depended on successful plasmid religation.

These results verified the functionality of the restriction–ligation panel and established that the recovered transformants originated predominantly from religated plasmid molecules rather than residual undigested plasmid.

### 2.2. PCR Detects Recombination

**Figure 1:**
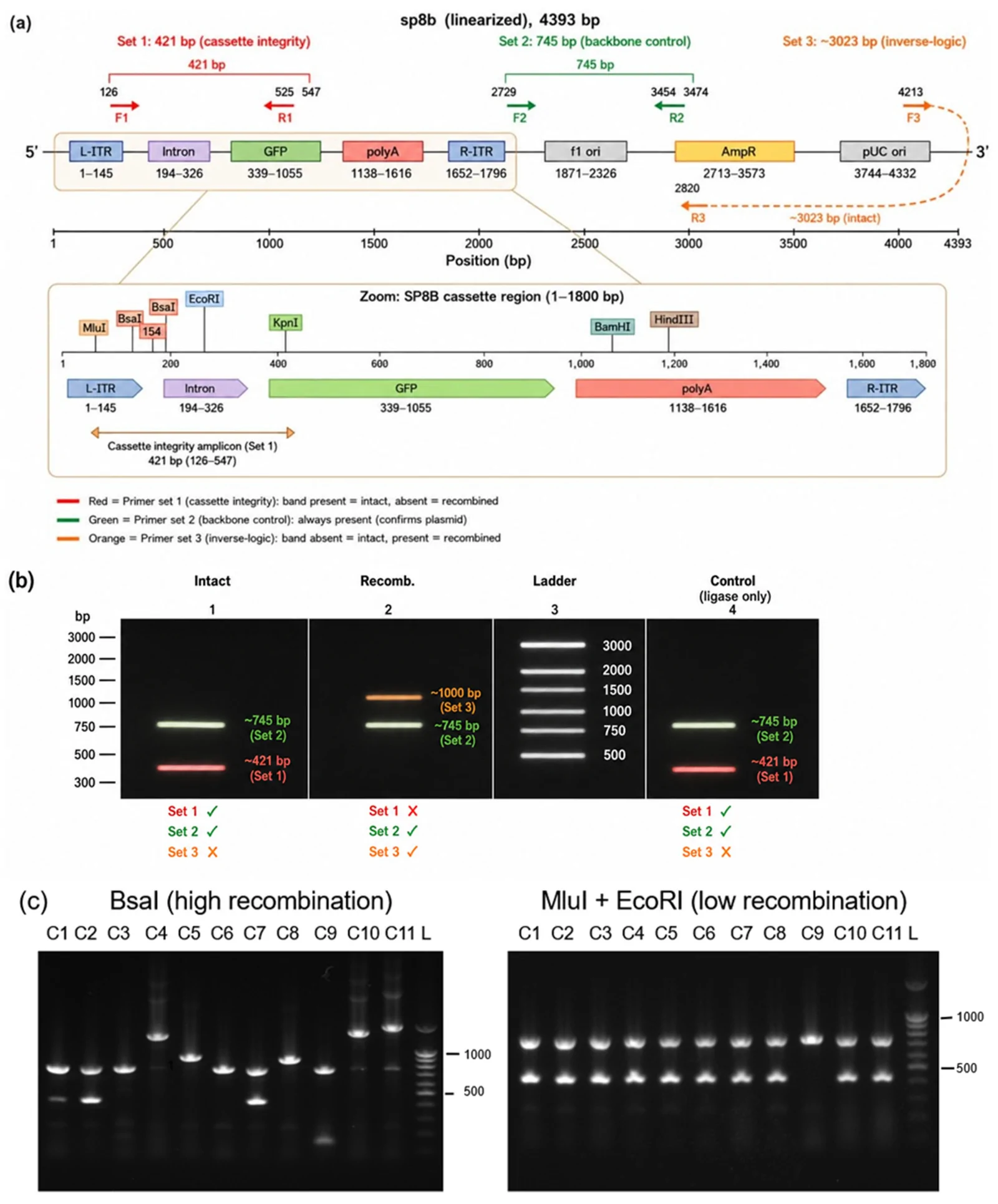
(**a**) Schematic representation of the linearized sp8b plasmid showing the binding positions and expected amplicon sizes of the three primer pairs used for plasmid integrity analysis: Set 1 (red, 421 bp, cassette integrity), Set 2 (green, 745 bp, backbone control), Set 3 (orange, ~3023 bp circular, inverse-logic). The lower panel shows a zoomed view of the ITR-flanked cassette region with restriction sites. (**b**) Representative agarose gel schematic with 4 lanes (Intact, Recombined, Ladder, Control) showing the expected band patterns for each primer set. (**c**) Example of agarose gel electrophoresis of high recombinant rate restriction (BsaI) and low recombinant rate restriction (MluI + EcoRI). Lanes C1–C11: amplicons from different colonies; Lane L: 100 bp DNA ladder (100, 200, 300, 400, 500, 600, 700, 800, 900, 1000 and 1500 bp).

Colony PCR using the two-primer scoring system revealed two classes of transformants. Intact clones produced the expected 421 bp cassette integrity amplicon together with the 745 bp backbone control amplicon. In contrast, recombinant clones retained the backbone control amplicon but showed either loss of the 421 bp cassette amplicon or the presence of an amplicon of unexpected size, consistent with structural rearrangement of the expression cassette (Figure 1).

The observed PCR patterns were reproducible across independent biological replicates. Notably, in a small number of recombinant clones, the backbone control amplicon also differed from its expected size. In contrast, all colonies recovered from the ligase-only control yielded the expected amplicons for both primer pairs, indicating that structural rearrangements were associated with constructs subjected to restriction digestion and religation rather than with the ligation reaction alone.

The frequency of plasmid rearrangements observed in the study substantially exceeded the 5–15% incidence of spontaneous ITR mutations reported during routine propagation of AAV plasmids [16,17]. These findings indicate that restriction digestion followed by religation increases plasmid instability compared with standard propagation conditions and are consistent with the hypothesis that introducing a ligation junction in close proximity to an AAV ITR promotes recombination during bacterial propagation [3,18].

Among the three *E. coli* strains evaluated, NEB Stable and Stbl3 exhibited comparable recombination frequencies, whereas TOP10 consistently showed higher levels of plasmid rearrangement. Because the primary objective of this study was to evaluate the effect of ligation junction position rather than host strain, all subsequent quantitative analyses were performed using the NEB Stable strain.

### 2.3. Recombination Increases with Cloning-Site Proximity to the ITR

**Figure 2:**
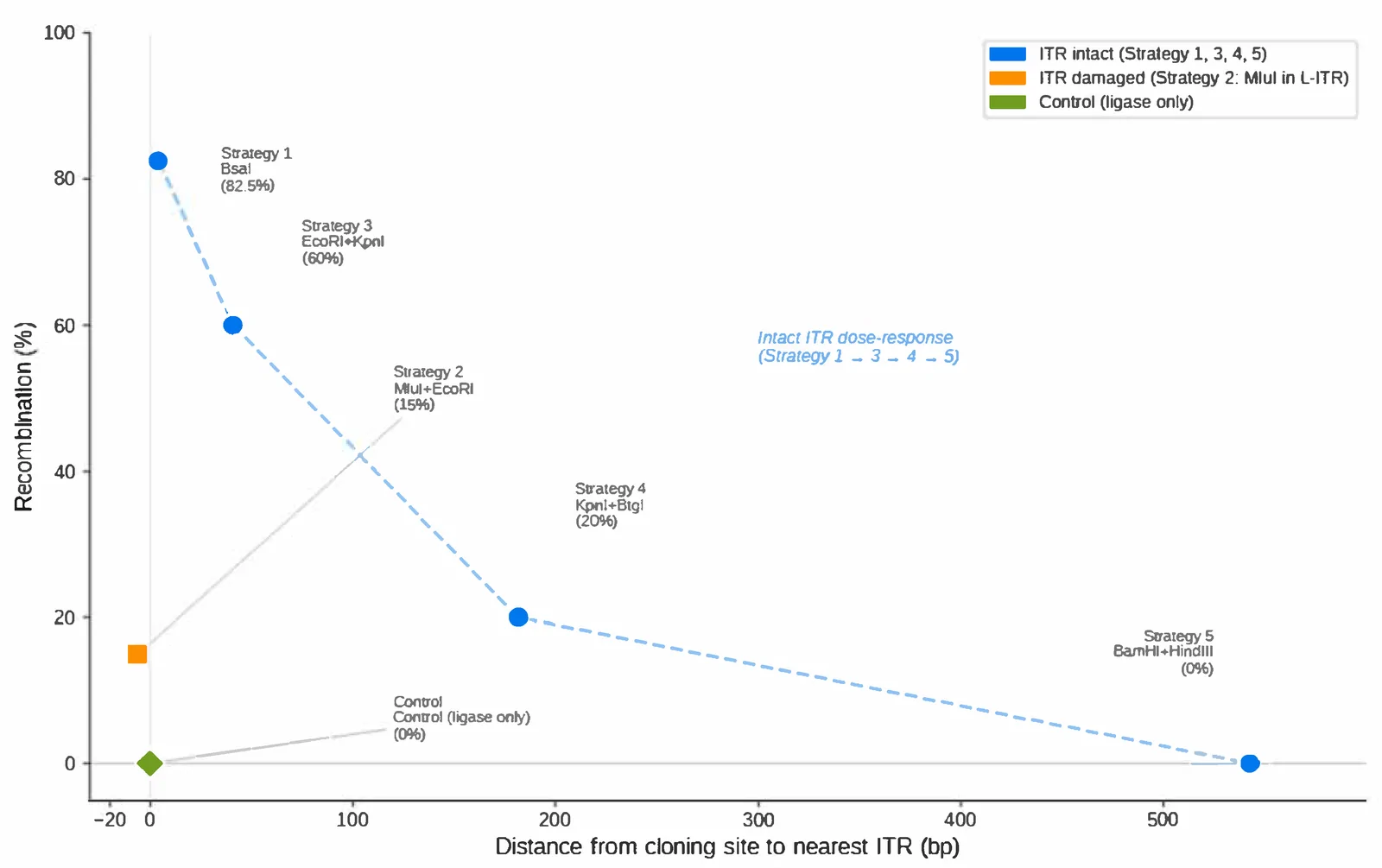
Distance-dependent plasmid recombination frequency.

Among the four strategies in which both ITRs remained intact, the frequency of recombinant clones decreased progressively as the distance between the ligation junction and the nearest ITR increased (Figure 2, Table 1). Strategy 1 (BsaI), in which the junction was located 4 bp from the L-ITR, yielded 82.5% recombinant clones. Increasing this distance to 41 bp (Strategy 3) reduced the recombination frequency to 60%, whereas a distance of 182 bp (Strategy 4) resulted in only 20% recombinant clones. No recombinant clones were detected when the ligation junction was positioned 543 bp from the nearest ITR (Strategy 5). These data demonstrate a strong inverse relationship between ligation-junction proximity to the nearest intact ITR and plasmid stability, indicating that junction position is a major determinant of recombination frequency during bacterial propagation.

Strategy 2 differed from the remaining constructs because digestion by MluI occurred within the L-ITR itself, disrupting the palindromic sequence before religation. Despite generating the ligation junction closest to the L-ITR, this construct exhibited only 15% recombinant clones, substantially lower than the 82.5% observed for Strategy 1, in which the L-ITR remained intact (Table 1).

This observation indicates that proximity of the ligation junction to the ITR alone is insufficient to account for the high recombination frequencies observed in the remaining constructs. Instead, preservation of an intact ITR appears to be required for the strong distance-dependent increase in recombination. Thus, although Strategy 2 generated the shortest junction-to-ITR distance of all constructs, disruption of the ITR markedly reduced plasmid rearrangement.

The ligase-only control produced exclusively intact plasmids, indicating that ligation alone did not induce detectable rearrangements. Likewise, reactions containing a single restriction enzyme (MluI, KpnI, or HindIII) together with DNA ligase did not exhibit rearrangement frequencies above background levels. Together, these controls demonstrate that elevated recombination was associated with constructs in which double digestion generated a new ligation junction.

### 2.4. De Novo NGS Assembly Confirms L-ITR-Initiated Deletion

To independently validate the PCR-based scoring and characterize the molecular structure of the recombination events, six clones from the BsaI strategy (Strategy 1) were subjected to whole-plasmid next-generation sequencing and de novo assembly. The results are summarized in Table 2.

The assembled recombinant plasmids ranged from 1863 to 2796 bp in length, corresponding to net deletions of 1597–2530 bp relative to the 4393 bp parental plasmids. The entire expression cassette—chimeric intron, GFP, and hGH polyA—together with the R-ITR was deleted in recombinant clones, while the backbone elements (AmpR, pUC ori) and the L-ITR were retained. The approximate deletion boundaries identified in individual recombinant plasmids are summarized in File S1, Figure S3. This indicates L-ITR-initiated deletion; recombination initiates at the L-ITR palindromic hairpin and extends unidirectionally through the entire cassette and R-ITR into the backbone, removing all intervening sequence. The recombinant plasmids contained novel junction sequences that were absent from the parental sp8b plasmid, consistent with deletion-associated DNA rejoining. Relative to the reference plasmid annotation, most recombinant molecules retained the annotated L-ITR while lacking the annotated R-ITR and the intervening expression cassette. This reproducible architecture demonstrates that the rearrangements are non-random, although the circular nature of the plasmid precludes inference of the direction or initiation site of the recombination event. The largest structural rearrangement was observed in clone M7 (2189 bp), which lost both ITRs and the entire cassette, in clone M7 (2189 bp), which lacked both AAV ITRs together with the entire expression cassette, retaining only the bacterial backbone containing the ampicillin resistance gene and the ColE1/pUC origin of replication.

The structural information from whole-plasmid sequencing was used to design an additional PCR assay (primer set 3; Methods, Section 4.7) for independent validation of the recombinant architectures. The forward primer binds within the pUC origin (nt 4213) and the reverse primer within the AmpR region (nt 2820). In the intact circular plasmid, these primers are separated by an approximately 3023 bp fragment spanning the pUC origin, L-ITR, expression cassette, R-ITR, f1 origin, and AmpR region, which was not efficiently amplified under the PCR conditions used. In contrast, recombinant plasmids containing deletion of the expression cassette generated amplification products of approximately 560–1500 bp, enabling rapid identification of sequencing-defined recombinant architectures.

### 2.5. Cloning into an ITR-FREE Plasmid Does Not Produce Recombinated Variants

To separate the contribution of the cloning junction itself from that of the AAV ITRs, four independently constructed barcoded libraries were built on the ITR-free variant of the sp8b plasmid and analyzed by long-read whole-plasmid sequencing. Two libraries were generated via Golden Gate cloning (SGC and SGR) and two via Gibson Assembly (SGIBC and SGIBR). Each pair carried either a CMV promoter (333 bp) or a DR1 (255 bp) insertion and comprised more than 2000 barcoded clones. De novo assembly of each library recovered a single dominant, high-coverage contig corresponding to the expected full-length plasmid, ranging from 4268 to 4354 bp. No alternative, shortened contigs were assembled, indicating that recombinant molecules were individually rare and structurally heterogeneous within the pooled clone populations. To capture and quantify these rare events, recombination was evaluated directly at the single-read level. Analysis revealed low recombination frequencies across all four libraries: 2.46% for SGC and 1.58% for SGR (Golden Gate), compared to 1.95% for SGIBC and 2.10% for SGIBR (Gibson Assembly). Across the libraries, 163 reads were classified as recombinant at the ≥300 bp threshold, with internal deletions being the most frequent class, followed by inversions and a minor fraction of junction deletions (Table 3).

**Figure 3:**
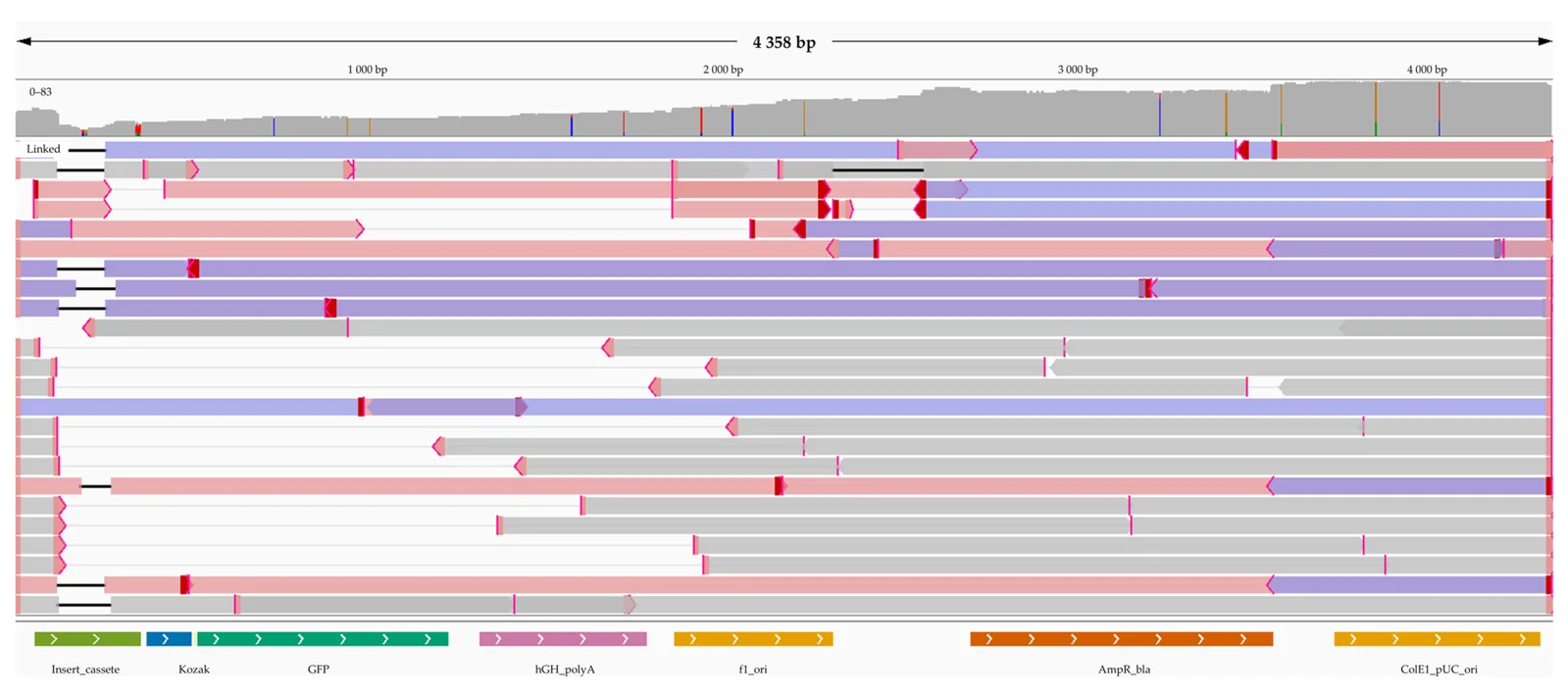
Representative recombination events in the ITR-free SGC library. Oxford Nanopore reads from the SGC library (Golden Gate assembly, CMV insert) mapped to their 4358 bp reference and visualized in IGV. The individual long reads exhibit diverse structural rearrangements, including junction deletions, internal deletions, and inversions, which are highlighted by black arrows. Grey bars represent aligned read segments, and supplementary alignments originating from the same single DNA molecule are connected by white thin horizontal lines. Horizontal black lines within the reads mark deleted reference spans. Red arrowheads and reversed arrow orientations highlight segments in the opposite strand direction, serving as the signature of an inversion. The colored tracks below the alignment panel show annotated plasmid features.

Notably, recombination frequencies observed across all four ITR-free sp8d libraries (1.58–2.46%) were substantially lower than those detected in the ITR-containing sp8b constructs with comparable or greater junction-to-ITR distances (e.g., 60% at 41 bp and 20% at 182 bp in Strategy 3 and 4). This indicates that neither the cloning method itself (Golden Gate vs. Gibson Assembly) nor the specific insert sequence (CMV vs. DR1) is sufficient to explain the high recombination rates observed in the ITR-containing backbone, and that the presence of an intact AAV ITR is the principal driver of the distance-dependent instability described above. Representative examples of these discrete and compound structural variants are visualized in Figure 3.

## 3. Discussion

### 3.1. Mechanistic Interpretation

The distance-dependent increase in plasmid recombination observed in this study is consistent with the current model of AAV ITR instability in *E. coli*, in which the palindromic ITR folds into a hairpin structure that is recognized and processed by the SbcCD nuclease complex, followed by RecBCD-mediated DNA end processing and deletion formation during DNA repair. These mechanisms have been proposed to underlie the instability of plasmids containing intact AAV ITRs during bacterial propagation [3,16].

When the cloning junction is positioned immediately adjacent to an intact ITR, the newly generated ligation site lies within the region most susceptible to ITR-associated DNA processing. Consequently, recombination events initiated at or near the ITR are more likely to propagate through the ligation junction and disrupt the inserted sequence or the downstream expression cassette. In contrast, increasing the distance between the ITR and the cloning junction spatially separates the inserted sequence from the region of highest instability. Under these conditions, ITR-associated processing may still occur, but it is less likely to extend far enough to affect the inserted fragment, thereby preserving cassette integrity. This phenomenon is analogous to, but mechanistically distinct from, the origin-proximity effect described by Radukic et al., in which the ITR located closest to the bacterial origin of replication exhibits the highest instability [3]. In contrast, the variable investigated here is the distance between the cloning junction and the nearest intact ITR. Whole-plasmid sequencing further supports this model. Most recombinant plasmids retained the L-ITR while deleting the downstream expression cassette together with the R-ITR, producing a highly reproducible deletion architecture. Although the sequencing data do not directly identify the site at which recombination is initiated, the recurrent retention of the L-ITR is consistent with DNA processing initiated at or near the intact L-ITR.

Additional support is provided by Strategy 2, in which the MluI restriction site lies within the L-ITR itself. Cleavage within the palindromic hairpin reduced the recombination frequency to 15%, despite generating the ligation junction closest to the ITR of all constructs tested. This observation indicates that physical proximity alone is insufficient to explain the elevated recombination frequencies observed for the remaining constructs and instead suggests that preservation of the intact ITR secondary structure is required for efficient recombination. This interpretation agrees with current models of AAV ITR biology and bacterial processing of palindromic DNA [3,16,19].

An additional factor may contribute to the exceptionally high recombination frequency observed for the BsaI construct. Golden Gate assembly relies on repeated cycles of restriction and ligation, and a fraction of plasmid molecules may transiently retain single-strand nicks at newly formed ligation junctions. When such nicks occur in close proximity to an intact ITR, they could increase accessibility of the surrounding DNA to bacterial repair pathways, thereby enhancing structural instability. Although this hypothesis remains speculative and requires direct experimental validation, it provides a plausible explanation for why the BsaI construct, in which the ligation junction is located only 4 bp from the L-ITR, exhibited the highest recombination frequency.

Finally, RecA-independent recombination involving palindromic DNA structures and repeated sequences has previously been described, and genome-wide analyses of bacterial plasmids have demonstrated that repeated DNA elements represent recurrent hotspots of structural instability [20,21]. The novel junction sequences identified in the recombinant plasmids are therefore compatible with repair mechanisms involving microhomology-mediated DNA rejoining or slipped-misalignment during repair synthesis, although the precise molecular pathway cannot be determined from the present data alone.

The factors influencing ITR integrity and stability are multi-factorial, and cloning-site-to-ITR distance is one important dimension of a broader landscape. The bacterial strain used for propagation is a major contributor: different *E. coli* strains carry distinct recombination and repair pathways (RecA, SbcCD, RecBCD) that differ in their capacity to process palindromic DNA structures, and strain choice can itself introduce mutations or rearrangements in ITRs during propagation [3]. Additional factors include growth temperature, plasmid copy number, insert sequence composition, and culture conditions, all of which can modulate the rate and spectrum of ITR-associated rearrangements [20,21]. Thus, the distance effect observed here should be considered as one component of the broader multifactorial determinants of AAV-MPRA plasmid stability.

In the context of AAV-based MPRAs, where the library is packaged into virus in a single pooled step, clones lost or rearranged during bacterial propagation cannot be recovered, and the delivered viral library is the recombination-filtered subset of the original design. Our finding that recombination frequency scales with cloning-site-to-ITR distance provides an actionable design parameter—one that can be controlled at the plasmid construction stage—to improve the integrity of AAV vector genomes entering the packaging pipeline.

It is important to acknowledge that the present study operates at the plasmid level: we quantify recombination during bacterial propagation and characterize the molecular structure of rearranged plasmids, but we do not directly measure downstream AAV packaging efficiency, viral genome content retention, or transgene expression in mammalian cells. The correlation between insert-to-ITR proximity, AAV expression efficacy, and genome content retention remains to be established experimentally. Nonetheless, the link between plasmid integrity and vector quality is well supported by the existing literature: ITR truncation and cassette deletion are known to reduce packaging yield and in vivo potency [3,16,19], and the recombination events we characterize—which delete the entire reporter cassette and one or both ITRs—are precisely the events that would produce defective viral genomes. Establishing the quantitative relationship between plasmid-level recombination frequency and AAV vector titre, full-capsid ratio, and transgene expression is a natural next step that would bridge the gap between plasmid design and gene-delivery outcomes.

### 3.2. Implications for AAV-MPRA Library Design and Study Limitations

The high frequency of ITR-associated plasmid rearrangements observed in this study has important implications for the construction of AAV-based MPRA libraries. In pooled MPRA experiments, recombination occurring during bacterial propagation affects not only the recovery of individual plasmid clones but also the integrity of the library as a quantitative measurement tool. Deletion or rearrangement of a regulatory element disrupts the linkage between the tested sequence and its associated barcode, resulting in incorrect assignment of regulatory activity and increasing experimental noise. Furthermore, because the probability of recombination depends on the position of the cloning junction relative to the ITR rather than on the inserted sequence itself, library composition becomes systematically biased before viral production [10,22]. Constructs cloned closest to the ITR are preferentially lost, reducing the effective complexity of the library and altering the relative representation of regulatory elements. In the context of AAV-MPRA, this loss is irreversible because only plasmids that survive bacterial propagation are packaged into viral particles [9,10]. Consequently, the delivered library represents a recombination-filtered subset of the intended construct collection. Our data demonstrate that when the ligation junction is located only 4 bp from the ITR, more than 80% of plasmids undergo rearrangement even in the recombination-deficient NEB Stable strain propagated at 30 °C, indicating that ITR-proximal cloning strategies can substantially compromise library integrity.

The observed distance dependence suggests several practical recommendations for the design of AAV-MPRA vectors. Whenever possible, the cloning junction should be positioned as far from the nearest ITR as permitted by vector architecture. In our experimental system, rearrangement frequency decreased from 82.5% at a junction-to-ITR distance of 4 bp to 20% at 182 bp, while no recombinant plasmids were detected when the nearest ITR was located 543 bp away. These results suggest that distances exceeding approximately 500 bp provide substantial protection against recombination, whereas distances below 200 bp remain associated with considerable plasmid instability. Although cleavage within the ITR itself markedly reduces recombination frequency, this strategy disrupts the essential ITR structure required for AAV genome replication and packaging and is therefore unsuitable for vector construction. The routine quality control of MPRA libraries should include junction-specific PCR assays before large-scale library amplification, viral packaging, or transfection. The multiplex PCR assay developed in the present study, together with the sequencing-derived validation assay, provides a rapid and inexpensive approach for identifying structurally rearranged plasmids before downstream experiments.

Several limitations of the present study should be considered. The distance range examined (4–543 bp) clearly demonstrated a monotonic relationship between ITR proximity and recombination frequency, although additional intermediate distances would enable more precise modelling of this relationship. Quantitative estimates were derived from analysis of 25–40 colonies per construct across two independent biological replicates and therefore should be interpreted as robust estimates of relative recombination frequencies rather than precise population parameters.

## 4. Materials and Methods

### 4.1. Plasmid Backbone

The reference plasmid used in this study was the 4393 bp pAAV-GFP MPRA reporter plasmid (sp8b), derived from pAAV2-CMV-GFP-BGHpA (Addgene #190239; Addgene, Watertown, MA, USA). The plasmid contains two AAV2 serotype ITRs flanking the reporter cassette. The expression cassette is organized as follows: AAV2 left ITR (L-ITR; nt 1–145), chimeric intron (nt 194–326), Kozak sequence (nt 333–338), GFP coding sequence (nt 339–1055), human growth hormone polyadenylation signal (hGH polyA; nt 1138–1616), and AAV2 right ITR (R-ITR; nt 1652–1796). The plasmid backbone additionally contains an f1 origin (nt 1871–2326), the ampicillin resistance gene (*bla*; nt 2713–3573), and the ColE1/pUC origin of replication (nt 3744–4332). The complete annotated plasmid sequence is provided in File S1, Figure S1, and Sequence S1. A multiple cloning site (MCS) is located between the AAV2 L_ITR_5′ and the chimeric intron. This region contains two BsaI restriction sites in opposite orientations, enabling Golden Gate assembly for MPRA downstream applications.

### 4.2. Construct Panel

To investigate whether the distance from the cloning site to the nearest ITR determines recombination frequency, we generated a panel of constructs by double restriction-enzyme digestion of sp8b followed by religation. Five restriction strategies were selected to generate ligation junctions at defined distances from the nearest ITR. The following restriction enzyme combinations were used:

1. BsaI. BsaI recognition sites are located at nt 154–160 and 175–181 and generate cleavage sites at nt 149–153 and 182–186. Following ligation, the resulting junction is positioned 4 bp downstream of the L-ITR boundary (nt 145). The L-ITR remains structurally intact.
2. MluI + EcoRI. MluI recognizes nt 139–144, while EcoRI recognizes nt 186–191. Cleavage by MluI occurs within the L-ITR, resulting in disruption of the ITR secondary structure before ligation. This construct served as a control for direct ITR damage.
3. EcoRI + KpnI. EcoRI and KpnI recognize nt 186–191 and nt 327–332, respectively. Ligation produces a junction located 41 bp downstream of the L-ITR while preserving an intact ITR.
4. KpnI + BtgI. KpnI and BtgI recognize nt 327–332 and nt 337–342, respectively. The resulting ligation junction is positioned 182 bp from the L-ITR, with the ITR remaining intact.
5. BamHI + HindIII. BamHI and HindIII recognize nt 1080–1085 and nt 1104–1109, respectively. Ligation generates a junction located 935 bp from the L-ITR and 543 bp from the R-ITR, while preserving both ITRs.

This construct panel allowed assessment of plasmid stability as a function of ligation junction proximity to the nearest intact ITR.

### 4.3. Restriction Digestion and Religation

Restriction digestion and ligation were performed under cycling conditions analogous to those used for Golden Gate assembly. Each 30 µL reaction contained 1× T4 DNA Ligase Reaction Buffer with ATP (New England Biolabs, Ipswich, MA, USA), 100 ng of sp8b plasmid DNA, 10 U of each restriction enzyme (New England Biolabs, Ipswich, MA, USA), according to the construct design described above, and 1000 U of T4 DNA ligase (Biospecifika, Novosibirsk, Russia). A ligation-only control (positive control) containing T4 DNA ligase, but no restriction enzyme, was included in each experiment. Reactions were subjected to 25 cycles of 37 °C for 90 s and 16 °C for 180 s, followed by a final incubation at 16 °C for 20 min and enzyme inactivation at 75 °C for 10 min. To verify restriction enzyme activity under the reaction conditions, parallel negative control reactions were performed containing a single restriction enzyme in the absence of DNA ligase.

Following completion of the reactions, DNA was purified using VAHTS DNA Clean Beads (Vazyme, Nanjing, China) according to the manufacturer’s instructions. DNA concentration was determined using a Qubit Fluorometer (Thermo Fisher Scientific, Waltham, MA, USA) prior to bacterial transformation.

### 4.4. Bacterial Strains and Transformation

Purified ligation products 1 ng as well as 100 pg of positive control and 10 ng of each negative control were transformed into electrocompetent *E. coli* cells of three strains: TOP10 (Thermo Fisher Scientific, Waltham, MA, USA)—standard cloning strain, and recombination-deficient strains—Stbl3 (Thermo Fisher Scientific, Waltham, MA, USA), NEB Stable (New England Biolabs, Ipswich, MA, USA).

Electroporation was performed using a MicroPulser Electroporator (Bio-Rad Laboratories, Hercules, CA, USA) according to the manufacturer’s recommendations. Following transformation, cells were recovered in SOC medium at 37 °C for 45 min, then plated on LB agar supplemented with ampicillin (100 µg/mL) and incubated at 30 °C for 14–16 h. The reduced incubation temperature was selected to improve the stability of plasmids containing AAV ITRs, as lower growth temperatures have been reported to reduce replication-associated instability of palindromic DNA sequences during bacterial propagation [3,13,23].

Each transformation experiment was performed independently in duplicate.

### 4.5. PCR-Based Recombination Detection

Plasmid recombination was assessed by colony PCR using two primer pairs in a multiplex reaction.

The first primer pair (cassette integrity assay) consisted of the forward primer 5′-TTCCTGCGGCCGCACGCGTAC-3′ and the reverse primer 5′-GCACGCCGTAGGTCAGGGTGGT-3′. This primer pair spans the AAV expression cassette and generates a defined-size amplicon from intact plasmids. Structural rearrangements affecting the cassette were identified by the absence of amplification or by the presence of an amplicon differing from the expected size. The second primer pair (backbone control assay) consisted of the forward primer 5′-TCCGTGTCGCCCTTATTCCC-3′ and the reverse primer 5′-GAGGGCTTACCATCTGGCCC-3′. This pair amplifies a fragment within the AmpR/ColE1-pUC backbone region and served as an internal control for plasmid presence.

Multiplex PCR was performed using the Biolabmix PCR Master Mix (Biolabmix, Novosibirsk, Russia) according to the manufacturer’s instructions. Amplification consisted of 30 cycles with an annealing temperature of 66 °C (20 s) and an 80 s elongation step (72 °C). Detection of amplicons was performed by agarose (1.2%) gel electrophoresis. A colony was classified as recombined when the backbone control amplicon was present while the cassette integrity amplicon was absent or differed from the expected size. Colonies producing the expected amplicons with both primer pairs were classified as intact.

Approximately 25–40 independent colonies were analyzed for each construct, including 15 colonies from the first biological replicate and 25 colonies from the second replicate. Colony counts from each transformation condition are summarized in File S1, Table S1. The recombination frequency was calculated as (number of recombinant colonies/total number of colonies analyzed) × 100.

### 4.6. Whole-Plasmid Sequencing and De Novo Assembly, Recombination Check

To independently validate the structural rearrangements detected by colony PCR, selected recombinant clones obtained from the BsaI construct (Strategy 1), which exhibited the highest recombination frequency, were cultured in LB medium supplemented with ampicillin, and plasmid DNA was isolated for whole-plasmid next-generation sequencing.

Sequencing libraries were prepared using the Raissol SG GM kit (Sesana, Moscow, Russia) and then sequenced using MiSeq Reagent Kit v2 for 300 cycles (Illumina, San Diego, CA, USA). Raw sequencing reads were evaluated using FastQC (version 0.11.9), followed by quality trimming and adapter removal using fastp (version 0.23.2). Filtered reads were assembled de novo using SPAdes (version 3.13.1) [24,25,26]. Sequencing reads were subsequently mapped to both the reference sp8b plasmid and the assembled contigs using Bowtie2 (version 2.3.5.1) to validate assembly accuracy and identify large-scale deletions or structural rearrangements [27]. Final contigs were aligned to the sp8b plasmid using BLASTn (version 2.9.0+) to identify structural rearrangements [28]. Novel junctions absent from the reference plasmid were identified to reconstruct the architecture of recombinant molecules.

To test directly whether AAV ITRs are responsible for rearrangements observed in ITRs-containing backbone, we constructed four plasmid libraries based on an ITR-free backbone (sp8d). The complete annotated sp8d plasmid sequence is provided in File S1, Figure S2, and Sequence S2. The plasmid pools were assembled via Golden Gate cloning (SGC, SGR) or Gibson Assembly (SGIBC, SGIBR) with one of two insertions: CMV or DR1 (File S1, Sequence S3 and S4). Each library was sequenced on an Oxford Nanopore platform. Raw reads were length-filtered using Chopper (version 0.13.0) to remove fragments shorter than 200 bp [29]. De novo genome assembly was performed using Flye (version 2.9.6) on the filtered long reads [30]. To improve consensus accuracy and correct systematic sequencing errors, the initial draft assembly was polished using Medaka (version 2.2.2) [31]. The final polished consensus sequence was aligned to the reference genome using minimap2 (version 2.31) with the -x asm5 preset [32]. Because each library comprised a pool of thousands of distinct barcoded clones, recombination events were quantified at the individual read level. Length-filtered reads were mapped to the reference genome using minimap2 with the -x map-ont preset, and alignment processing, sorting, and indexing were performed using SAMtools (version 1.24) [33]. To prevent origin-spanning reads of circular molecules from being misidentified as rearrangements, reads were additionally aligned to a head-to-tail doubled copy of each reference, rotated to place the cloned insert near the center. Reads with a primary plasmid alignment were retained for downstream analysis. Based on their alignment structure, reads were classified as either normal (full-length, collinear) or recombined. A read was classified as recombined if it contained a deletion, junction deletion, or inversion of ≥300 bp, requiring ≥ 200 bp of aligned flanking sequence on each side of the junction. Small fluctuations within the insert cassette were excluded to avoid assembly boundary wobble artifacts. The recombined fraction was calculated as the percentage of total plasmid-mapped reads.

### 4.7. PCR Validation of Sequencing-Defined Rearrangements

The structural information obtained from whole-plasmid sequencing was used to design an additional PCR assay for independent validation of recombinant plasmid architectures. The validation primer pair consisted of the forward primer 5′-CGCACGAGGGAGCTTCCAGGG-3′, which binds within the pUC origin at nt 4213, and the reverse primer 5′-ACCCACTCGTGCACCCAACTGA-3′, which binds within the AmpR region at nt 2820.

In the intact circular sp8b plasmid, these primers are oriented toward each other and generate a predicted ~3023 bp product spanning the pUC origin, L-ITR, expression cassette, R-ITR, f1 origin, and AmpR region. Due to the length of this fragment and the PCR extension conditions used, the intact plasmid was not efficiently amplified, and no detectable product was observed. In contrast, recombinant molecules containing deletions between the primer-binding sites generated shorter products (~560–1200 bp), enabling independent detection of the sequencing-defined structural rearrangements.

## Figures and Tables

**Table 1:** Recombination rates of different restriction-enzyme strategies on sp8b.

Strategy	Enzyme(s)	Cut Site(s) (nt)	Distance to ITR (bp)	ITR Integrity	% Normal Clones	% Recombinant Clones
1	BsaI (Type IIS)	150–153, 182–185	4 (L-ITR)	Intact	17.5	82.5
2	MluI + EcoRI	140–143, 186–191	−6 (L-ITR)	Damaged (MluI cuts inside L-ITR)	85	15
3	EcoRI + KpnI	187–190, 328–331	41 (L-ITR)	Intact	40	60
4	KpnI + BtgI	328–331, 338–341	182 (L-ITR)	Intact	80	20
5	BamHI + HindIII	1081–1084 1105–1108	543 (R-ITR) 935 (L-ITR)	Intact	100	0
Control	Ligase only (no enzyme)	—	—	Intact	100	0

**Table 2:** De novo NGS assembly of six clones from Strategy 1 (BsaI, 4 bp from L-ITR, 82.5% recombination).

Clone	Assembled Size (bp)	Net Change vs. Ref (bp)	L-ITR	R-ITR	Intron	GFP	hGH polyA	f1 Ori	AmpR	pUC Ori	Predicted Amplicon Primer Mix 3 (bp)
C1	2386	−2007	+	−	−	−	−	−	+	+	1016
C10	1990	−2403	+	−	−	−	−	−	+	+	620
M4	1863	−2530	+	−	−	−	−	−	+	+	493
M7	2189	−2204	−	−	−	−	−	−	+	+	818
M8	2796	−1597	+	−	−	−	−	+	+	+	1426
M13	2144	−2249	+	−	−	−	−	−	+	+	774

**Table 3:** Long-read analysis of the four ITR-free barcoded libraries. The recombined fraction is calculated as the percentage of plasmid-mapped reads carrying a deletion, junction deletion, or inversion ≥ 300 bp relative to the library’s respective reference. Event counts are given at the ≥ 300 bp threshold; a single read may contribute to more than one event type.

Clone	Assembled Full-length Contig, bp	Reads Mapped	Recombined Reads (≥300 bp)	% Recombined (≥300 bp)	Event Counts (Deletions/Junction Deletions/Inversions)
SGC	4354	2888	71	2.46	39/13/21
SGR	4305	3094	49	1.58	28/6/15
SGIBC	4332	1129	22	1.95	12/5/8
SGIBR	4268	998	21	2.10	12/4/7

## Data Availability

The raw data supporting the conclusions of this article will be made available by the authors on request.

## Supplementary Materials

The following supporting information can be downloaded at: https://www.mdpi.com/article/10.3390/ijms27177630/s1.
